# Wax Ester Synthase/Diacylglycerol Acyltransferase Isoenzymes Play a Pivotal Role in Wax Ester Biosynthesis in *Euglena gracilis*

**DOI:** 10.1038/s41598-017-14077-6

**Published:** 2017-10-18

**Authors:** Takuya Tomiyama, Kaeko Kurihara, Takahisa Ogawa, Takanori Maruta, Takumi Ogawa, Daisaku Ohta, Yoshihiro Sawa, Takahiro Ishikawa

**Affiliations:** 1 Department of Life Science and Biotechnology, Faculty of Life and Environmental Science, Shimane University, 1060 Nishikawatsu, Matsue, Shimane 690-8504 Japan; 2 Core Research for Evolutional Science and Technology (CREST), Japan Science and Technology Agency (JST), Chiyoda-ku, Tokyo, 102-0076 Japan; 3 Graduate School of Life and Environmental Sciences, Osaka Prefecture University, 1-1 Gakuen-chou, Nakaku, Sakai, Osaka, 599-8531 Japan

## Abstract

Wax ester fermentation is a unique energy gaining pathway for a unicellular phytoflagellated protozoan, *Euglena gracilis*, to survive under anaerobiosis. Wax esters produced in *E. gracilis* are composed of saturated fatty acids and alcohols, which are the major constituents of myristic acid and myristyl alcohol. Thus, wax esters can be promising alternative biofuels. Here, we report the identification and characterization of wax ester synthase/diacylglycerol acyltrasferase (WSD) isoenzymes as the terminal enzymes of wax ester production in *E. gracilis*. Among six possible *Euglena* WSD orthologs predicted by BLASTX search, gene expression analysis and *in vivo* evaluation for enzyme activity with yeast expressing individual recombinant WSDs indicated that two of them (EgWSD2 and EgWSD5) predominantly function as wax ester synthase. Furthermore, experiments with gene silencing demonstrated a pivotal role of both EgWSD2 and EgWSD5 in wax ester synthesis, as evidenced by remarkably reduced wax ester contents in EgWSD2/5-double knockdown *E. gracilis* cells treated with anaerobic conditions. Interestingly, the decreased ability to produce wax ester did not affect adaptation of *E. gracilis* to anaerobiosis. Lipid profile analysis suggested allocation of metabolites to other compounds including triacylglycerol instead of wax esters.

## Introduction

A wide variety of microalgae provide various useful biochemical compounds such as carotenoids, long chain unsaturated fatty acids, pigments, and polysaccharides^1^, and are also considered promising feedstock for biofuel as substitute for fossil fuels^2^. Regarding the resources for biodiesel fuel, some microalgae, such as *Chlorella*, *Chlamydomonas*, and *Nannochloropsis*, have a capability to accumulate triacylglycerol (TAG) as neutral lipids under nutrition-limiting conditions, such as under deficiency of nitrogen and phosphate^3,4^. Unlike these microalgae, a unicellular phytoflagellated protozoan, *Euglena gracilis*, has an ability to produce and accumulate wax esters instead of TAG under oxygen-limited conditions^5–7^. The wax esters produced in anaerobic *E. gracilis* cells are composed of saturated esters, mainly myristyl myristate (C28) and considerable amounts of C26 and C27, and thus consist of medium-chain fatty acids and alcohols ranging from C10 to C18 with the majority being the 14:0 carbon chains accounting for 44% of total wax ester contents^5–7^. Owing to its low freezing point and a high Cetane number (66.2)^8^, myristic acid (C14:0) has greater potential as a drop-in jet fuel than other medium-length algal fatty acids such as palmitic acid (C16:0) and stearic acid (C18:0).

The metabolic pathway of anaerobic wax ester synthesis in *E. gracilis* has been identified and designated “wax ester fermentation” owing to a concomitant generation of ATP without any energy loss during the wax ester production^5,7^. In the wax ester fermentation process, fatty acids are synthesized *de novo* in mitochondria by utilizing acetyl-CoA, not malonyl-CoA, as primer and C2 donor, stemming from pyruvate gained after cytoplasmic glycolysis step^9^. The acyl-CoA is successively reduced *via* reversible enzymatic steps of β-oxidation by participation of a unique medium-chain preferring *tran*-2-enoyl CoA reductase instead of acyl-CoA dehydrogenase^10^, and the resultant acyl-CoA is exported to the endoplasmic reticulum and then converted into fatty alcohol by fatty acyl-CoA reductase (FAR)^11,12^. Finally, wax esters are produced by the esterification of fatty acyl-CoA and fatty alcohol, catalyzed by wax ester synthase (WS) or acyl-CoA:fatty alcohol acyltransferase^13^.

Enzymes exhibiting activity of wax ester synthesis have been identified and characterized from a wide range of organisms, and have been classified into two main groups. The first group belongs to typical WSs, which exhibit activity only for wax ester synthesis, identified from mammals, such as human and mouse^14^, and higher plants, such as jojoba^15^ and petunia^16^. The mammalian WSs show efficient catalytic activities toward medium chain acyl-CoAs from C12 to C16 in length and fatty alcohols shorter than C20, as the acyl donor and acceptor, respectively. In contrast, WS from jojoba showed activity with both saturated and monounsaturated acyl-CoAs ranging from C14 to C24, and showed highest activity against fatty alcohols with C18:1 and C18:2^15^. The second group comprises bifunctional enzymes with both WS and acyl-CoA:diacylglycerol acyltransferase (DGAT) activities, which was first identified and characterized from *Acinetobacter calcoaceticus* ^17^. The wax ester synthase/diacylglycerol acyltransferase (WS/DGAT), abbreviated as WSD hereafter, is distributed widely in prokaryotes, but is not genetically related to the first group WSs and known TAG synthesis enzymes, such as DGAT1/2 families and phospholipid:diacylglycerol acyltransferases^17,18^. The WSD utilizes a broad range of acyl-CoAs and fatty alcohols from C12 to C20 in length^17^. Apart from bacteria, the orthologous genes for WSD have also been identified in *Arabidopsis thaliana* and it has been demonstrated that the AtWSD1 predominantly plays a vital role for wax ester production in the stem^19^.

Previously, Teerawanichpan and Qiu^13^ identified WS in *E. gracilis*, that showed significant similarity to jojoba WS, and confirmed that recombinant WS has the ability to produce myristyl myristate and other wax ester molecules through heterologous co-expression systems in yeast cells with *E. gracilis* FAR. However, there is still no convincing physiological evidence whether the *E. gracilis* WS plays a major role in wax synthesis *in vivo* in response to anaerobic conditions. Recently, we performed a comprehensive gene expression analysis in *E. gracilis*, and identified six contigs with significant similarity to WSD^20^. In this study, we characterize the putative WSDs using heterologous expression systems in yeast cells, and then evaluated their physiological contributions toward wax ester synthesis in *E. gracilis* cells using gene silencing method. We show that the WSDs are indeed involved in wax ester formation *in vivo* under anaerobic conditions, and discuss their physiological significance.

## Results

### Putative WSD orthologs in *E. gracilis*

**Figure 1:**
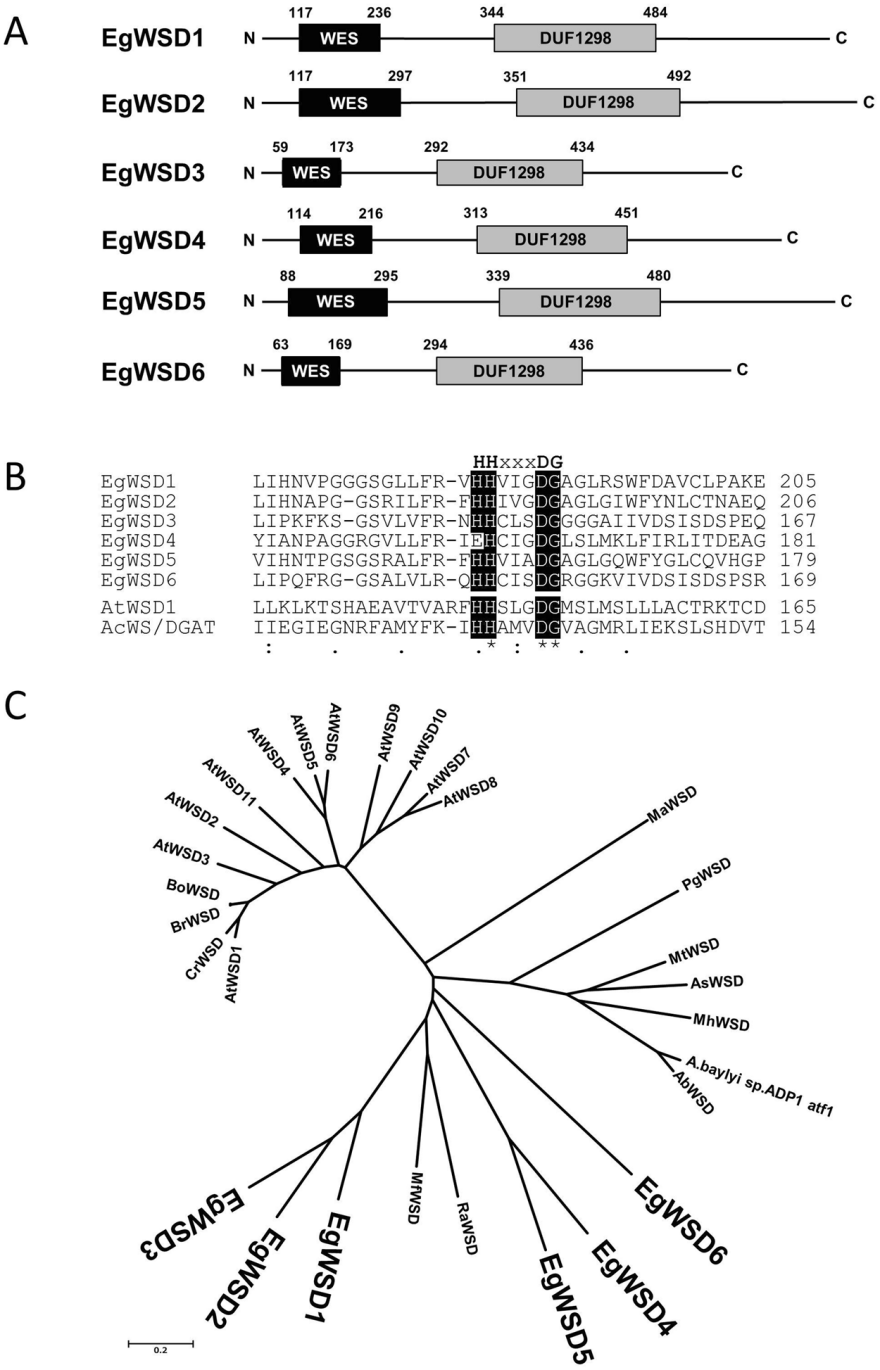
Primary structure of WSD isoforms in *E. gracilis*. (**A**) Molecular organization of six WSD isoforms. Designations of putative domains were based on Pfam domain database: WES, wax ester synthase-like acyl-CoA acyltransferase domain; DUF1298, domain of unknown function. (**B**) Comparison of putative active site residues of WSDs from *Euglena* (EgWSD1-6, LC069357-LC069364), Arabidopsis (AtWSD1, At1G72110) and *Acinetobacter calcoaceticus* ADP1 (AcWS/DGAT, AF529086) using the ClustalW program. Amino acids matching the putative active site motif HHxxxDG are shaded in black. (**C**) Phylogenetic analysis of the six WSDs from *Euglena* and proteins related to WSD from other organisms. Phylogenetic trees were constructed with full-length WSD amino acid sequences using the neighbor-joining (NJ) method using the MEGA 6.0 software (http://www.megasoftware.net/). Sequence alignments were assembled by the ClustalW algorithem version 2.1. Abbreviations and UniProt accession numbers except for EgWSDs are as follows: At, *Arabidopsis thaliana* (AtWSD1-11, Q93ZR6, Q9C7H4, F4IU14, Q9M3B3, Q9M3B2, Q9M3B1, Q94CK0, Q9FFE8, Q9FK89, Q9FK04, Q5KS41); Bo, *Brassica oleracea var. oleracea* (A0A0D3APY8); Br, *Brassica rapa* (A0A078CR28); Cr, *Capsella rubella* (R0IEG2); Ab, *Acinetobacter baumannii* (A0A077GKS2); As, *Amycolicicoccus subflavus* DQS3-9A1 (F6EKR7); Mh, *Marinobacter hydrocarbonoclasticus* (A3RE50); A.baylyi sp.ADP1 aft, *Acinetobacter baylyi* sp. ADP1(Q8GGG1); Mt, *Mycobacterium tuberculosis* (P9WKC7); Ra, *Rhodococcus aetherivorans* (A0A1Q8I8D0); Mf, *Myxococcus fulvus* 124B02 (A0A0F7DYG7); Ma, *Mucor ambiguus* (A0A0C9N7W4); Pg, *Photobacterium ganghwense* (A0A0J1HAU3).

To identify potential orthologs whose products are expected to show wax ester synthesis activity, an amino acid sequence of *A. calcoaceticus* WSD was used as a query for BLASTX search against *E. gracilis* RNA-Seq database constructed previously (DRA accession number: SRP060591)^20^. Six components with relatively high similarity to *A.calcoaceticus* WSD ranging from 43.3% (7.1% identity) to 64.8% (20.2% identity) were selected as the potential candidates encoding *Euglena* WSD (Table 1). We designated them as EgWSD1 to EgWSD6. With a search against the Pfam domain database (http://pfam.xfam.org/)^21^, we identified wax ester synthase-like acyl-CoA acyltransferase domain (PF03007) and DUF1298 domain (PF06974), the latter of which is a conserved domain of hypothetical plant proteins of unknown function, in the N- and C-terminal halves, respectively, of all six EgWSDs (Fig. 1A). In the former domain, a predicted active site motif responsible for ester bond^22^, HHXXXDG, is highly conserved in all EgWSDs, except for EgWSD4 whose first His residue is replaced by glutamate (Fig. 1B). These protein structures including the active site motif are well consistent with other WSDs from *A.calcoaceticus* and Arabidopsis. In addition, four EgWSDs, except for EgWSD5 and EgWSD6, were predicted to have one or two transmembrane domains, using TMHMM server V.2.0 (http://www.cbs.dtu.dk/services/TMHMM/), SOSUI program (http://harrier.nagahama-i-bio.ac.jp/sosui/), DAS-Transmembrane Prediction server (http://www.sbc.su.se/~miklos/DAS/tmdas.cgi), and TMpred (http://www.ch.embnet.org/software/TMPRED_form.html), expected to be membrane bound (Supplementary Fig. S1). Given the absence of obvious organellar targeting signals, the most likely location of EgWSDs, excluding EgWSD5 and EgWSD6, is likely to be the ER membrane, which is the main place of wax ester synthesis in *E. gracilis* ^7^. On the other hand, as for EgWSD5 and EgWSD6, prediction of their hydrophobicity varied depending on programs used, indicating they will be of ambiguous protein with relation to localization. Further observations using specific antibody will be needed for understanding their accurate localization in the future work. EgWSD orthologs were compared with various WSDs from bacteria and plants by generating phylogenetic tree based on ClustalW analysis (http://clustalw.ddbj.nig.ac.jp/) (Fig. 1C). The EgWSDs were rather closely related to bacterial WSD families, but not plant ones, and formed their own clades, suggesting they evolved uniquely.

### Expression of WSD orthologous genes in *E. gracilis*

**Figure 2:**
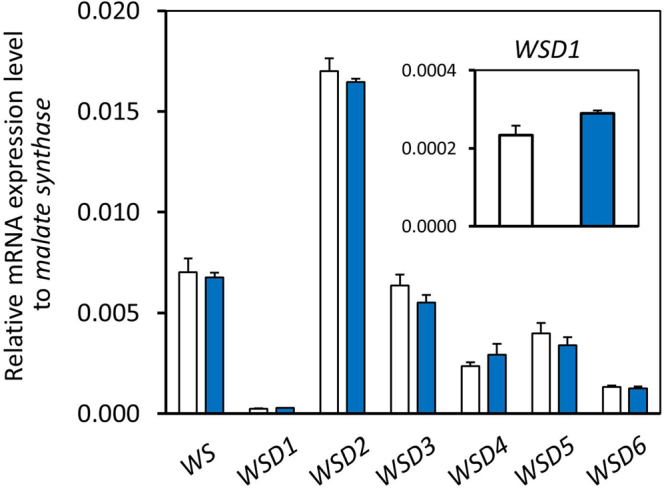
Quantitative expression analysis of *WS* and *WSD* genes in response to aerobic and anaerobic conditions. Total RNA was extracted from 7-d-old *Euglena* SM-ZK cells grown heterotrophically under normal growth conditions (white bar) and the cells anaerobically treated for 24 h (blue bar). Quantitative PCR analysis was performed to determine the expression levels of the indicated genes. Relative expression levels were normalized to malate synthase mRNA. Values are the mean ± SD of three independent measurements.

The active biosynthesis of wax ester in *E. gracilis* is evoked by encountering hypoxic conditions^5,6^. Previous comprehensive *E. gracilis* gene expression analysis showed that the expression levels of the six *WSDs* varied considerably (Table 1)^20^. We then performed qPCR analysis to anticipate which *EgWSD* would function predominantly in response to anaerobic conditions. As shown in Fig. 2, under ordinary aerobic conditions, the expression level of *EgWSD2* was markedly higher than that of other *EgWSDs* and *WS* genes. Following *EgWSD2*, the expression levels of *EgWSD3* and *EgWSD5* were ranked high, in that order. The expression level of *EgWSD1* gene was very low and almost negligible. Importantly, a comparative expression analysis of aerobic- and anaerobic-treated *E. gracilis* cells showed that gene expression levels in all *EgWSD* genes were not significantly altered by anaerobic treatment (Fig. 2). This result was well consistent with the previous RNA-Seq data that gene expression changes in predicted components involved in wax ester metabolism were not extensive or dynamic during the anaerobic treatment^20^.

### *In vivo* evaluation of wax ester synthesis activities of recombinant EgWSDs in yeast

**Figure 3:**
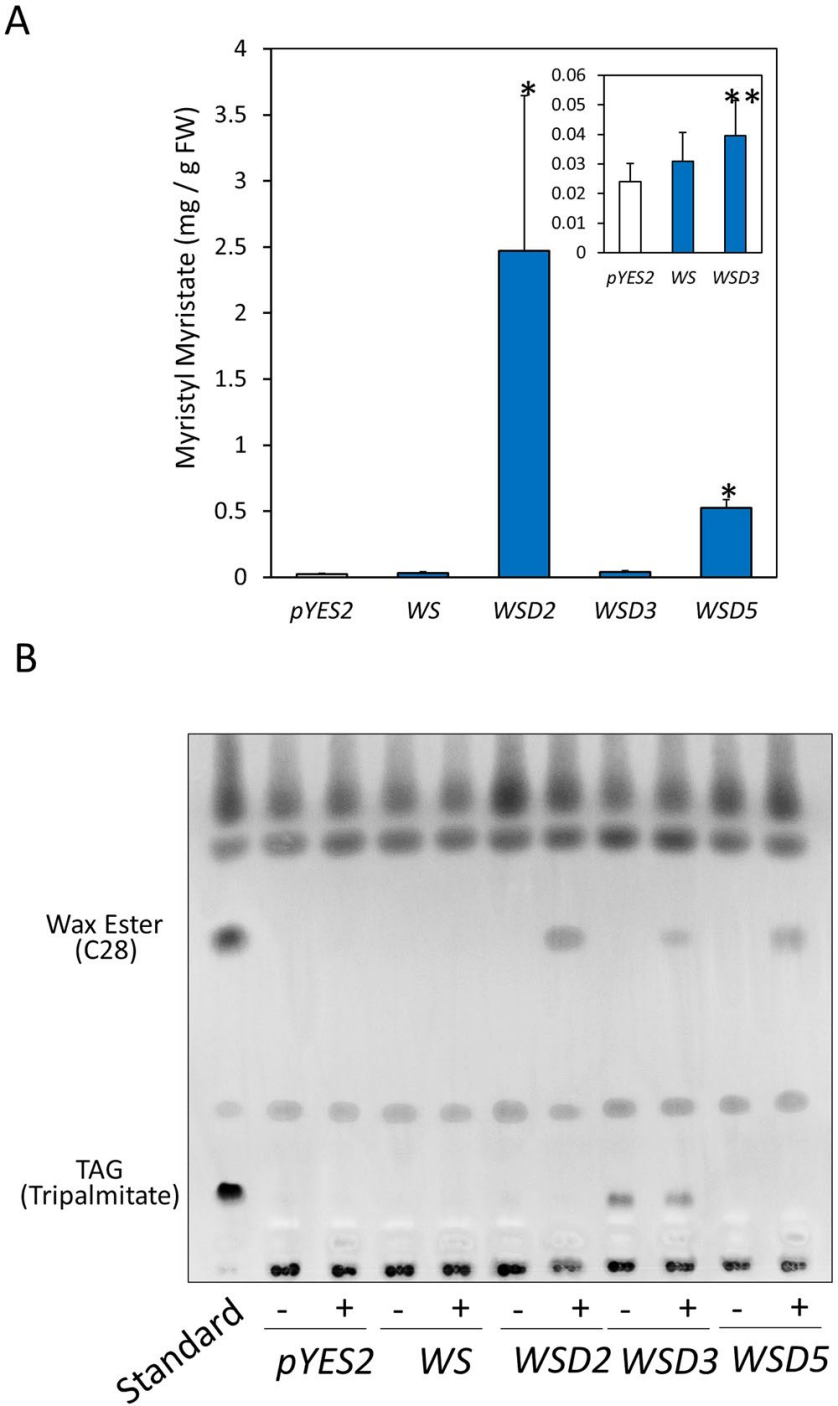
*In vivo* evaluation of wax ester synthesis activities of EgWSDs in yeast H1246 cells. (**A**) TAG synthesis-deficient yeast mutant transformed with an empty vector (pYES2), WS, and WSDs were grown to exponential phase and were incubated for 48 h after supplementation of 250 µM myristic acid and 1-tetradecanol. Myristyl myristate contents were determined and quantified by GC-MS analysis. Asterisks denote statistically significant differences (*p < 0.01, **p < 0.05) compared with vector control (pYES2). Values are the mean ± SD of three independent experiments. (**B**) TLC analysis of whole yeast extracts from each transformed line supplemented with both 250 μM myristic acid and 1-tetradecanol (+), and 250 μM myristic acid only (−). Lipids were extracted from each transformed yeast, and separated on a Silica gel 60 plate as described in Materials and Methods section.

**Figure 4:**
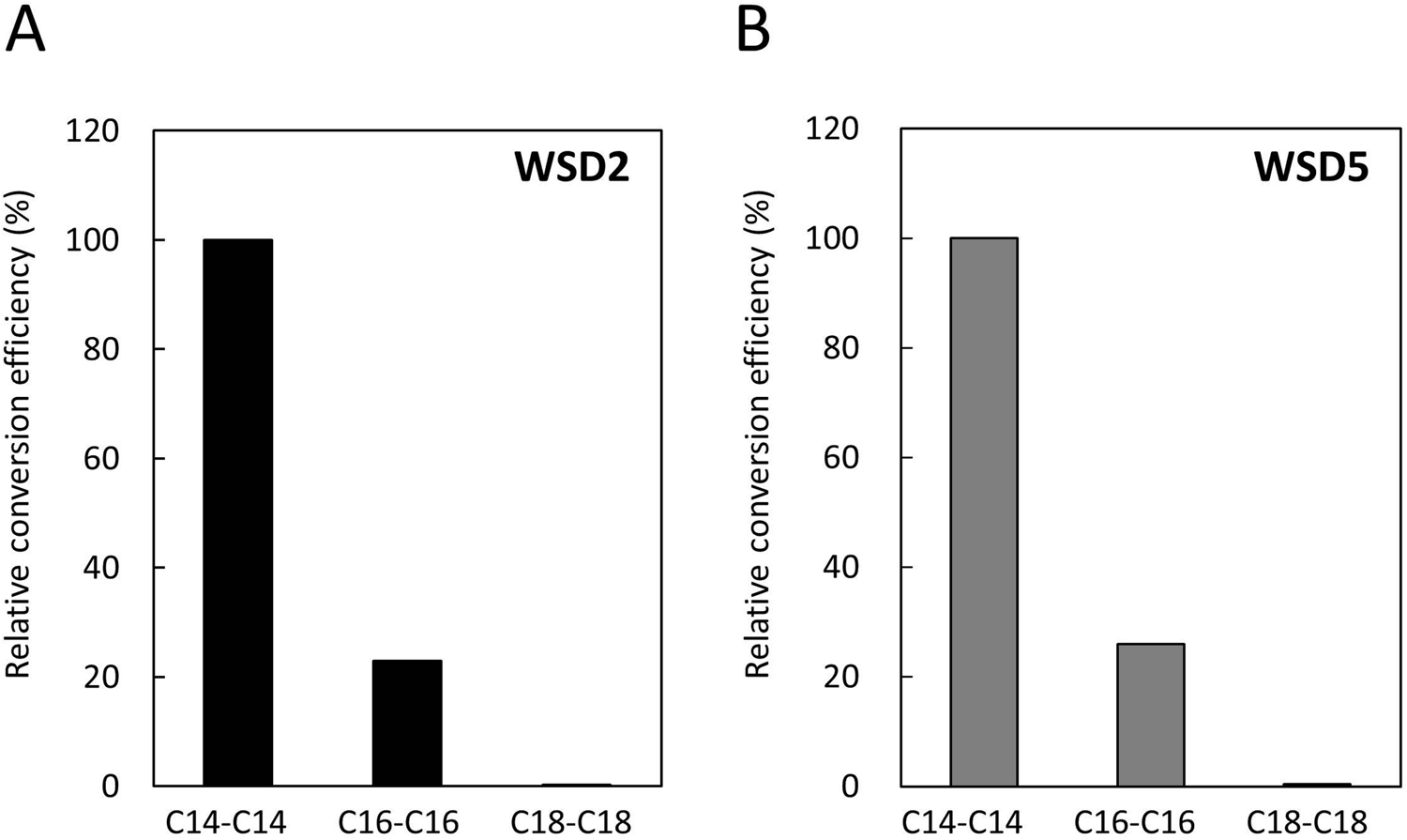
Substrate specificity of WSD2 and WSD5. The analysis was performed using transformed yeast H1246 expressing recombinant WSD2 (**A**) and WSD5 (**B**). Yeast cells were grown to exponential phase and were incubated for 24 h after supplementation of 250 µM fatty acids (myristic acid, palmitic acid, stearic acid) and fatty acid alcohols (1-tetradecanol, 1-hexadecanol, 1-octadecanol). The resultant wax ester contents were determined and quantified by GC-MS analysis. The results are expressed as relative efficiency on the basis of myristyl myristate (C14-C14) production. Values are means of two independent experiments.

To verify whether enzymes produced from the *EgWSD2*, *EgWSD3*, *EgWSD5*, and *WS* genes do indeed exhibit the wax ester synthesis activities, they were heterologously expressed in *Saccharomyces cerevisiae* strain H1246 which was disrupted at four key TAG synthesis related genes, *ARE1/2*, *DGA1*, and *LRO1* ^23^. This strain showed no diacylglycerol acyltransferase activity, resulting in neutral lipid-deficient phenomenon and providing suitable background for evaluating recombinant WSD activities. The transformed yeast H1246 cells harboring the recombinant pYES2 with individual *EgWSDs* and *WS* genes were induced by the addition of D-galactose. Using a semi-quantitative RT-PCR, we confirmed that all transformed yeast H1246 cells were successfully expressed in individual transgenes with almost the same expression strength after the D-galactose induction (data not shown). The cells were further incubated for 48 h after supplementation of 250 μM myristic acid and myristic alcohol, and then C28 contents were determined and quantified by gas chromatography-mass spectrometry (GC-MS) analysis. Both *EgWSD2*- and *EgWSD5*-introduced yeasts exhibited remarkable levels of C28 accumulation (approx. 2,500 and 500 μg/gFW, respectively) compared with the empty vector control (25 μg/gFW) (Fig. 3A). In *EgWSD3*-introduced yeast, C28 accumulation was significant, but severely impaired compared with *EgWSD2*- and *EgWSD5*-introduced yeasts. In contrast, *EgWS*-introduced yeast showed C28 levels equivalent to the empty vector control. As shown in Fig. 3B, a thin-layer chromatography (TLC) analysis was supported the quantitative results from GC-MS, and also showed no detectable accumulation of TAG even under the medium without providing myristic alcohol, except for *EgWSD3*-introduced yeast cells which showed significant spots corresponding to TAG standard under the medium conditions with/without myristic alcohol. The results suggested that at least two major WSDs, EgWSD2 and EgWSD5, were enzymatically specific toward wax ester synthesis, but not utilize diacylglycerol as substrate. On the other hand, EgWSD3 seems to be bifunctional enzyme like bacterial WSD families. To evaluate substrate specificity of EgWSD2 and EgWSD5, we added various fatty acids and fatty alcohols with carbon lengths C16 and C18. As shown in Fig. 4, both enzymes readily utilized C14 fatty acid and C14 alcohol as substrates, showed low activity with C16 substrates, and almost negligible reaction toward C18 substrates. The results clearly indicated the reaction of EgWSD2 and EgWSD5 with a high specificity toward shorter chain length fatty acids and alcohols.

### Effect of EgWSD gene silencing on wax ester production

**Figure 5:**
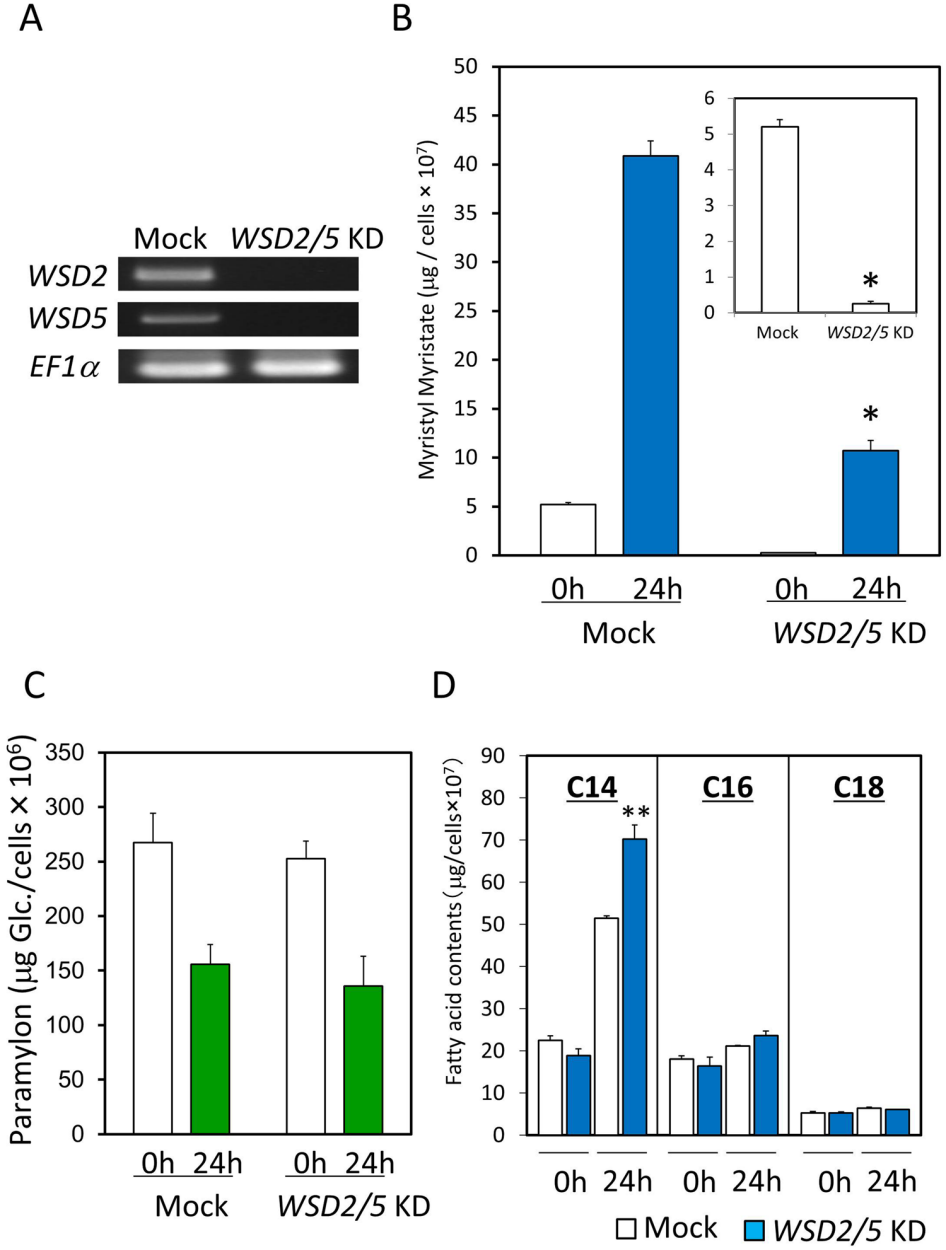
Effect of WSD2 and WSD5-double gene knock down (*WSD2/5* KD) on wax ester fermentation. (**A**) Verification of gene silencing by RT-PCR. RT-PCR was carried out with total RNA from *Euglena* cells in which dsRNA was introduced. Mock cells electroporated without dsRNA. (**B**) Influence of *WSD2/5* KD on myristyl myristate production under anaerobic conditions. *Euglena* cells grown to stationary phase were anaerobically treated for 24 h and then collected for wax ester measurement as described in Material and Method section. The inset shows the results for 0 h in detail. Asterisk denotes statistically significant differences (*p < 0.01) compared with mock control. Values are the mean ± SD of three independent experiments. (**C**) Influence of *WSD2/5* KD on paramylon content and consumption under anaerobic condition. Paramylon contents were finally determined by the phenol-sulfuric acid method using glucose solution as a standard. Values are the mean ± SD of three independent experiments. (**D**) Influence of *WSD2/5* KD on fatty acid contents under anaerobic condition. Asterisks denote statistically significant differences (**p < 0.05) compared with mock control. Values are the mean ± SD of three independent experiments.

To evaluate the physiological significance of EgWSDs in the synthesis of wax ester in *E. gracilis* cells, we temporarily suppressed their expression using RNA-mediated interference. A double-stranded RNA (dsRNA) synthesized from part of the *EgWSD* sequences was introduced into *Euglena* cells by electroporation. Initially, we tried to silence individual EgWSDs and EgWS. The effect of silencing on expression of individual targeted genes was subsequently confirmed by RT-PCR (Supplementary Fig. 2A). However, none of the individual gene-silenced cell lines showed significant difference in wax ester accumulation after 24 h-anaerobic treatment, indicating functional compensation each other (Supplementary Fig. 2B). Notably, the C28 level in EgWSD2-silencing cells under aerobic growth conditions was significantly lower than that in other cell lines, indicating that EgWSD2 would be the dominant enzyme for wax ester synthesis at least under ordinary aerobic conditions. Subsequently, based on the results of gene expression analysis and recombinant EgWSD activities shown in Figs 2 and 3A, respectively, we chose two WSDs, *EgWSD2* and *EgWSD5*, as dominant WSD genes expressed in *E. gracilis* cells, and tried to silence them simultaneously. As a result, the double-knockdown (double-KD) cell line showed considerable loss of wax ester accumulation under anaerobic conditions (Fig. 5), indicating that they are substantial wax ester synthases. It is also worth noting that the wax ester content in the *WSD2/5* double-KD cell line was negligible in aerobiosis, whereas that in mock-control cells was still detectable (Fig. 5B inlet). The *WSD2/5* silencing did not affect cell growth and viability even under anaerobic conditions at least for 48 h (data not shown). We then measured paramylon and fatty acid contents to confirm the effect of gene silencing on their productivity and consumption. As shown in Fig. 5C, the amounts of paramylon did not differ significantly between *WSD2/5*-silenced cell lines and mock control cells before and after anaerobic treatments. In addition, the amount of myristic acid in *WSD2/5* silencing cells rather increased by approximately 35% under anaerobic conditions (Fig. 5D). These facts clearly indicate that the ability for paramylon and d*e novo* fatty acid synthesis is not affected by WSD gene silencing, and the reaction process of wax ester synthesis is not a limitation factor for metabolic regulation of these metabolites during wax ester fermentation.

### Lipid profile analyses of EgWSD2/5 double-KD cells

**Figure 6:**
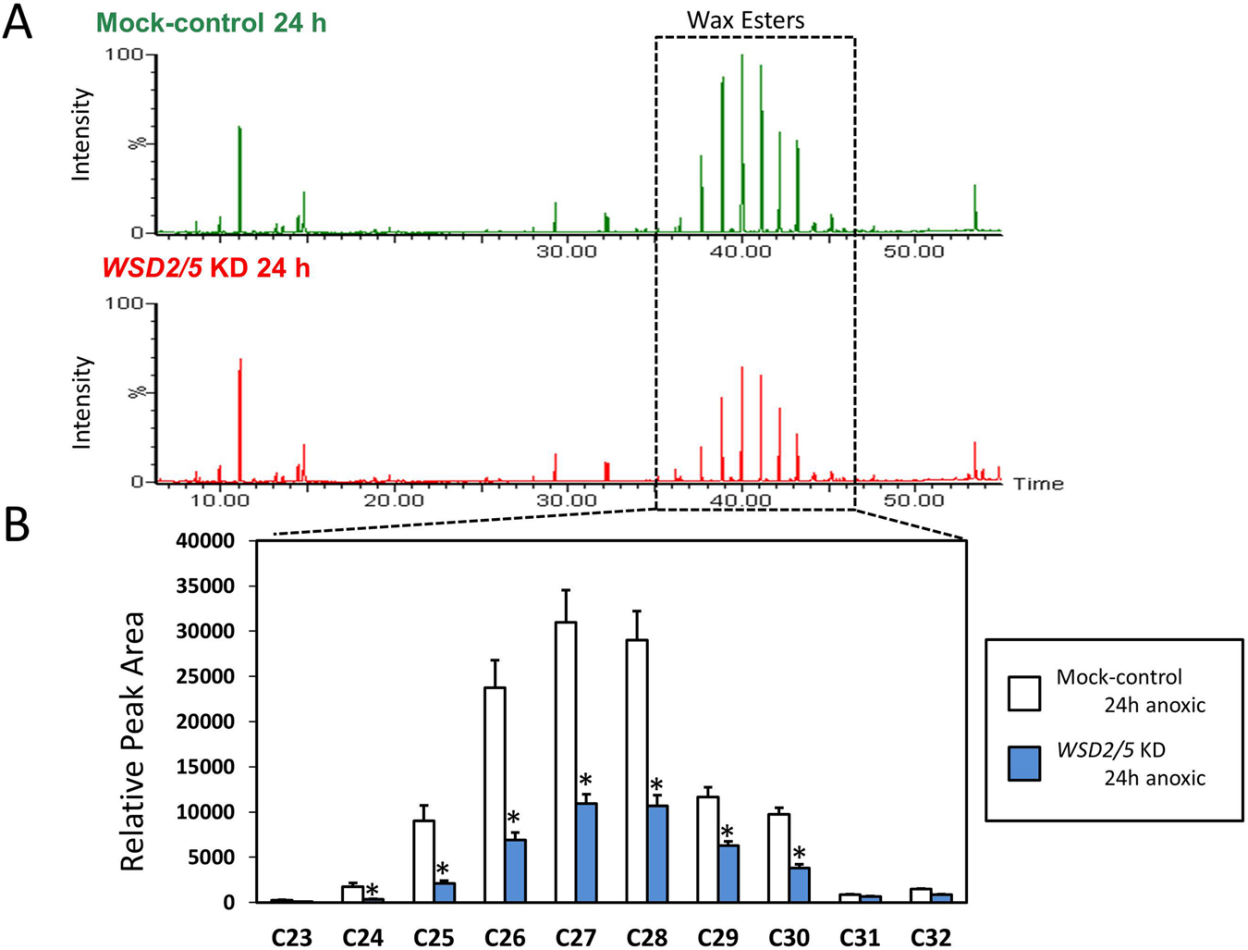
GC-TOF/MS-based lipid profiling between mock control and WSD2/5-KD. Aerobically grown *Euglena* cells were placed under anaerobic conditions for 24 h. The *Euglena* cells were collected at 0 h and 24 h. Total lipid was extracted from the *Euglena* cells and dried using a centrifugal evaporator. The non-methylesterified lipid fractions prepared from the mock control and WSD2/5-KD cells were analyzed by GC-TOF/MS. Wax ester contents were determined by a GC-TOF/MS-based profiling method as described by Furuhashi *et al*.^24^. (**A**) Representative GC-TOF/MS total ion chromatograms (TIC) of mock-control and WSD2/5-KD *Euglena* cells treated with anaerobic conditions for 24 h. (**B**) Comparison of relative amount of wax ester molecules between mock control and WSD2/5-KD. The data was processed based on the region enclosed by the dashed line. Asterisks indicate statistically significant differences between mock-control and WSD2/5-KD *Euglena* cells (means ± SD, *n* = 3; biological replicate, Student t-test, *p < 0.05).

**Figure 7:**
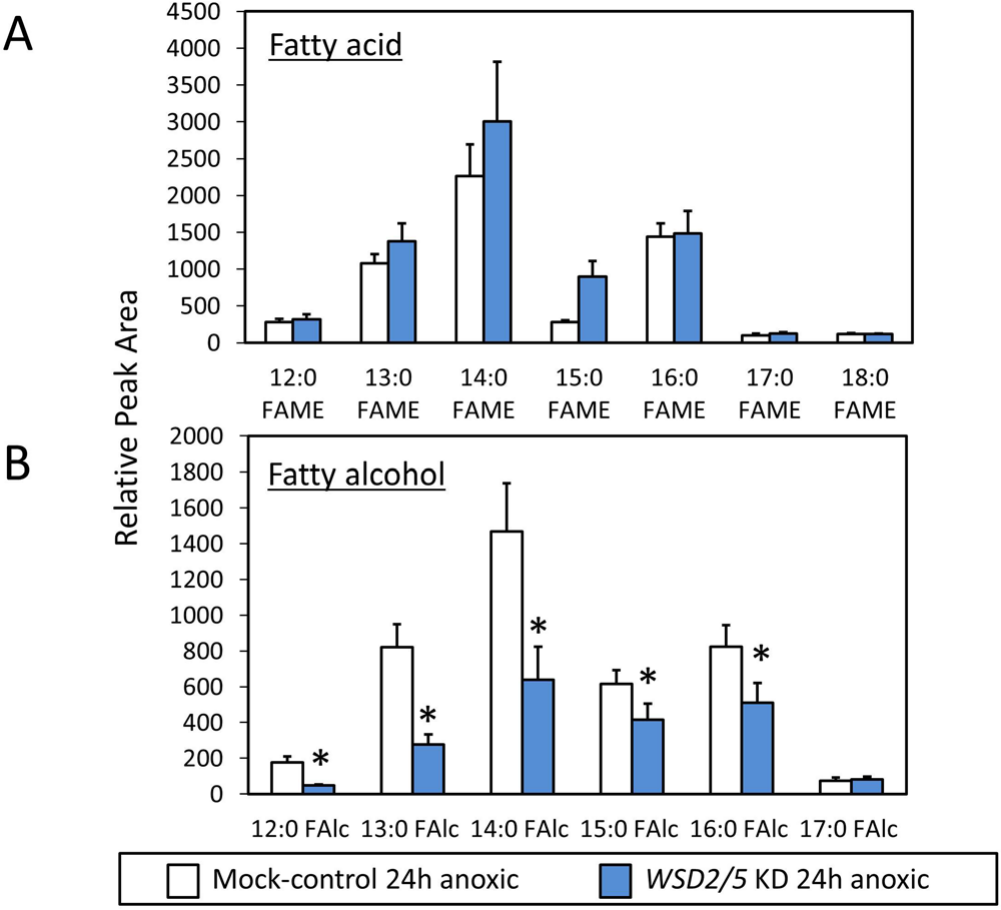
GC-TOF/MS-based profiling of the ester-linked fatty acids and fatty alcohols. Aerobically grown *Euglena* cells were placed under anaerobic conditions for 24 h. The *Euglena* cells were collected at 0 h and 24 h. Total lipid was extracted from the *Euglena* cells and dried using a centrifugal evaporator. Sodium methoxide in methanol was added to the dried lipid pellet to transesterify the ester-linked lipids, followed by trimethylsilylation; then the methyl esters and the TMS derivatives were subjected to GC-TOF/MS analysis^24^. Asterisks indicate statistically significant differences between mock-control and WSD2/5 KD cells in each time points (means ± SD, *n* = 3; biological replicate, Student t-test, *p < 0.05).

**Figure 8:**
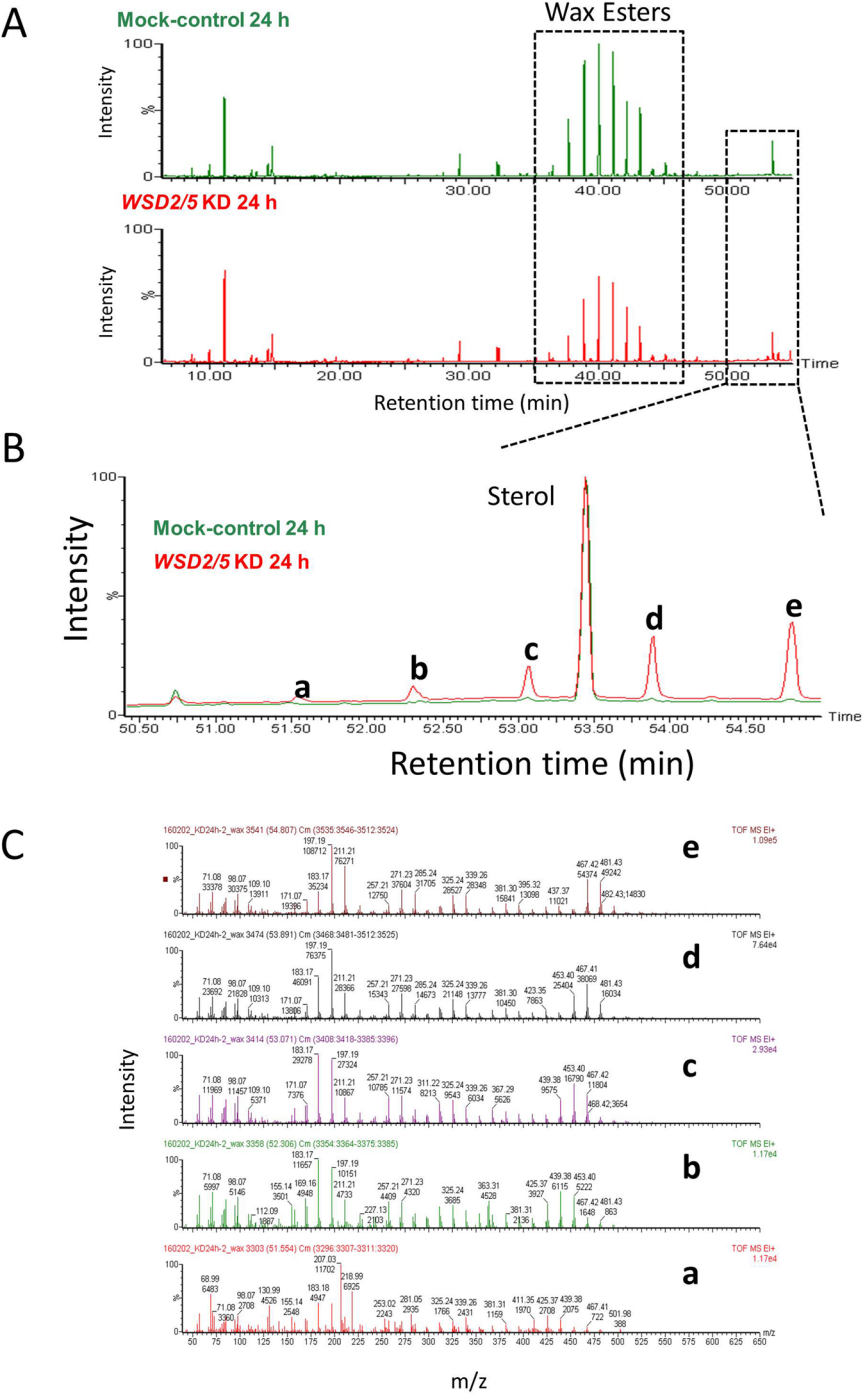
Comparison of GC-TOF/MS-based lipid profiling between mock-control and WSD2/5KD. The non-methylesterified lipid fractions prepared from the mock control and WSD2/5KD cells were analyzed by GC-TOF/MS. (**A**) Representative GC-TOF/MS total ion chromatograms (TIC) of mock-control and WSD2/5-KD *Euglena* cells treated with anaerobic conditions for 24 h. (**B**) Chromatogram enlarging the area surrounding the dashed line. Letters a-e indicate unique peaks observed in the TIC of the WSD2/5-KD at 24 h. (**C**) Mass spectra of the unique peaks observed in the TIC of the WSD2/5-KD at 24 h.

**Figure 9:**
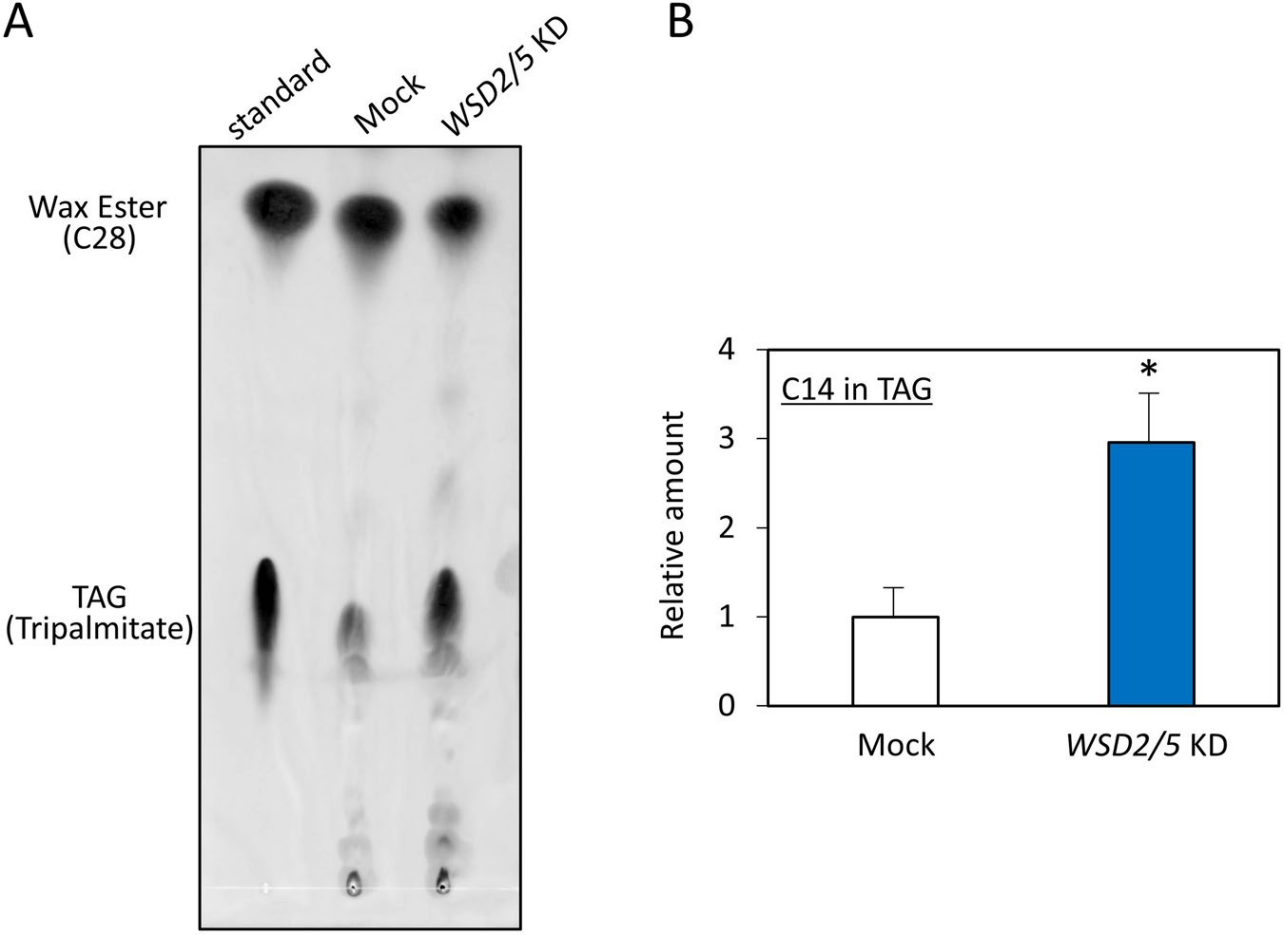
TLC analysis of wax ester and TAG in WSD2/5 KD cells treated anaerobically for 24 h. (**A**) Total lipids were extracted from mock and WSD2/5 KD cells, and separated on a Silica gel 60 plate as described in the Materials and Methods section. (**B**) Comparison of C14 contents consisting of TAG molecules in the mock and EgWSD2/5 KD cells. TAG spots were scraped, eluted, and trans-esterified to fatty acid methyl esters (FAME). The FAME was analyzed as the lipid compositions by GC/MS. Values are the mean ± SD of three independent experiments. Asterisks denote statistically significant differences (*p < 0.05) compared with the mock control.

We now have a simple question what the fatty acids generated in EgWSD2/5 double-KD cells under anaerobic condition were converted into in substitution for wax esters. To verify the clue, we performed detailed lipid profile analyses according to Furuhashi *et al*.,^24^. As shown in Figs 6 and 7A, the relative amount of wax esters, including C28, was significantly low in EgWSD2/5 double-KD cells, whereas that of fatty acids did not differ between the silenced cells and mock control cells, obviously supporting the result obtained in Fig. 5. Interestingly, when we analyzed the relative amounts of fatty alcohols, the EgWSD2/5 double-KD cells had decreased levels of not only C14 alcohols but also other fatty alcohol molecules (Fig. 7B). In addition, by comparing a lipid profile between mock control and the silencing cells, we observed a remarkable difference in a certain part with high molecular weight at a retention time over 50 min, as shown in Fig. 8A and B. Their mass spectrum indicated that they were some kind of TAG molecules (Fig. 8C). Hence, TAG levels in the EgWSD2/5 double-KD cells were compared by conventional TLC analysis. As expected, the amount of TAG molecules in the silencing cells increased, in contrast to the decreased wax esters content (Fig. 9A). TAG, which co-migrated at the same position as the standard TAG, was then scraped, eluted and trans-esterified to fatty acid methyl esters (FAME). The FAME was analyzed as the lipid compositions by GC/MS. The relative amount of C14 in the EgWSD2/5 double-KD cells increased by approximately three-fold in the mock control cells, suggesting that some of free C14 molecules are allocated into TAGs, resulting in TAG accumulation (Fig. 9B).

## Discussion

*E. gracilis* is considered to rely on wax ester fermentation to survive anaerobic conditions^5,6^. Elucidating the metabolic processes is key to understanding the cellular physiology in absence of oxygen. Such studies provide valuable information regarding whether it is necessary for *E. gracilis* to synthesize wax esters for the express purpose of sustaining ATP production under anaerobic conditions. Furthermore, from a biofuel perspective, it is critical to understand functions of metabolic enzymes involved in wax ester production. Wax esters in *E. gracilis* consist mainly of C14 acids and C14 alcohols, and Teerawanichpan and Qiu^13^ previously reported WS as the enzyme catalyzing their formation, although its physiological significance at cellular level has not been assessed. In this study, based on our previous comprehensive gene expression analysis of *E. gracilis* ^20^ and homology search for the enzyme that mediates wax ester formation in *A.calcoaceticus* ^17^, we predicted that EgWSD isoforms are the key enzymes catalyzing the last step of wax ester biosynthesis in this microalga. Several biochemical evidences discussed below revealed that this is certainly the case.

Firstly, a comprehensive gene expression analysis indicated that the expression levels of *EgWSD* genes were much higher than that of *EgWS* gene (Table 1, Fig. 2)^20^. In particular, genes that showed higher expression levels were *EgWSD2*, *EgWSD5*, and *EgWSD3*. Secondly, among the six WSD orthologs in *E. gracilis*, gene silencing of *EgWSD2* having the highest gene expression level exhibited marked deficiency in the accumulation of C28 under aerobic condition (Supplementary Fig. S2). Under anaerobic conditions, although silencing of individual genes including WS showed no significant difference in C28 accumulation, simultaneous silencing of both *EgWSD2* and *EgWSD5*, genes of two high ranks having high expression levels, exhibited significant decreases in C28 accumulation (Fig. 5B). This finding clearly indicated that EgWSD2 is the most dominant enzyme at least in aerobic wax ester production, and EgWSD5 might be the major anaerobic wax ester synthesis enzyme together with EgWSD2. The residual amount of C28 (approximately 30% in mock control cells), which was still present in the EgWSD2/5 double-KD mutants, indicated that there might be a contribution of other WSD isoforms like EgWSD3, which show significant activity for C28 synthesis (Fig. 3A). Thirdly, when EgWS and EgWSD were expressed in the TAG biosynthesis defective yeast mutant H1246^22^, both EgWSD2 and EgWSD5 showed conspicuous wax ester synthesis ability when supplemented with both myristic acid and myristic alcohol as the substrates; however, EgWS did not (Fig. 3A). Therefore, based on these results, we concluded that WSDs play a pivotal role in wax ester biosynthesis in *E. gracilis*.

**Table 1:** Summary of the homology search by BLASTX program for the wax ester synthase and wax ester synthase/acyl-CoA:fatty alcohol acyltransferase in *E. gracilis*. The FPKM values were quoted from our previous study^20^, and are averages of at least three independent experiments. Abbreviations are the following; AE, aerobic; AN, anaerobic.

Component ID	Abbreviation	Description	E-value	FPKM			Fold change	
				AE_0 h	AN_12 h	AN_24 h	12 h/0 h	24 h/0 h
comp25371_c1_seq. 1	EgWS	Probable long-chain-alcohol O-fatty-acyltransferase 3 in Arabidopsis thaliana_Q9FJ74|WAXS3_ARATH, identical to Euglena wax ester synthase	4.00E-13	1.414	1.182	1.289	0.84	0.91
comp29378_c0_seq. 1	EgWSD1	Putative diacyglycerol O-acyltransferase Mb3154c in Mycobacterium bovis_P0A651|Y3154_MYCBO	2.00E-14	2.953	2.262	2.871	0.77	0.97
comp17367 c0_seq. 1	EgWSD2	Putative diacyglycerol O-acyltransferase Mb0919 in Mycobacterium bovis_P67205|Y919_MYCBO	3.00E-14	41.802	35.502	36.134	0.85	0.86
comp31582_c0_seq. 2	EgWSD3	Putative diacyglycerol O-acyltransferase Rv1425/MT1468 in Mycobacterium tuberculosis_P71694|Y1425_MYCTU	1.30E-14	23.037	22.934	21.659	1.00	0.94
comp34975_c0_seq. 2	EgWSD4	Putative diacyglycerol O-acyltransferase Rv1760/MT1809 in Mycobacterium tuberculosis_O06795|Y1760_MYCTU	1.50E-07	6.14	6.712	7.021	1.09	1.14
comp33545_c0_seq. 1	EgWSD5	Putative diacyglycerol O-acyltransferase Rv1760/MT1809 in Mycobacterium tuberculosis_O06795|Y1760_MYCTU	3.00E-11	25.105	26.502	23.549	1.06	0.94
comp34299_c0_seq. 1	EgWSD6	Putative diacyglycerol O-acyltransferase Rv1760/MT1809 in Mycobacterium tuberculosis_O06795|Y1760_MYCTU	4.00E-37	7.984	6.055	5.757	0.76	0.72

In agreement with the catalytic activity of WSD1 in Arabidopsis^19^, the results that both EgWSD2 and EgWSD5 expressed in yeast strain H1246 showed significant activities toward wax ester synthesis, but not TAG synthesis, also suggested that EgWSDs do not exhibit DGAT activity *in vivo* (Fig. 3B). This fact indicated that WSD enzymes in *Euglena* and plants have different catalytic properties from those in prokaryotes because a representative WS/DGAT from *A.calcoaceticus* ADP1 restored TAG biosynthesis when expressed in the same yeast strain H1246^22^. This assumption is also supported by the fact that Arabidopsis WSD1 mutant showed marked suppression of wax ester accumulation^19^, whereas WS/DGAT-disrupted *Streptomyces* strain had lower TAG amount than WT strain^25^. Although biochemical evidence to explain the relationship between primary structure and enzyme activity of WSD is still limited, in contrast to WSD in *A.bayliyi* sp. ADP1^18^, EgWSD isoforms do not possess similarities to the acyl-CoA:glycerol-3-phosphate acyltransferase motifs in their primary amino acid sequences, supporting our conclusion that EgWSDs are not bifunctional enzymes like typical bacterial WSDs, and function as enzymes only for wax ester synthesis. As for substrate specificity of *Euglena* WSDs, in agreement with the composition of *Euglena* wax esters^7^, they preferably utilize C14 fatty acid and C14 alcohol (Fig. 4). In contrast, *Arabidopsis* WSD1 showed higher activity toward longer chain acyl acceptors like C24 or C28^19^. Consequently, WSDs from *Euglena* are likely novel enzymes specific for wax ester synthesis that recognize middle lengths of fatty acids and alcohols.

From the perspective of metabolic regulation from paramylon degradation to wax ester production in response to anaerobic conditions, there is now considerable evidence that paramylon accumulation and its degradation, and the following *de novo* fatty acid production were not affected by the suppression of wax ester synthesis, clearly indicating that the last reaction of the pathway is not a limiting step for the entire metabolic process. Although further experimental evidence needs to identify the crucial step of the regulation, it is at least suggested that conversion of pyruvate to acetyl-CoA by pyruvate:NADP^+^ oxidoreductase (PNO) is one of key processes considering that PNO functions in anaerobic conditions and its suppression caused serious cell death under the same conditions^9,26^. It is worth noting that fatty alcohol levels in EgWSD2/5 double-KD cells were significantly lower than those in the mock control, as indicated by lipid profile analysis (Fig. 7). It is likely that FAR cooperates with WSD for efficient wax ester synthesis. Regarding increasing TAG levels in EgWSD2/5 double-KD cells, incorporation of helpless fatty acids into non-toxic TAG might be a reasonable strategy to protect cells from high concentrations of free fatty acids generated under anaerobic conditions because they are considered to be potentially damaging to cell membrane^27^. As for TAG biosynthesis in *Euglena*, although it has never been reported so far, EgWSD3 may participate in TAG production due to its TAG synthesis activity. Or at least three putative contigs encoding DGAT (comp21783_c0_seq. 1, comp24776_c1_seq. 1, comp30904_c0_seq. 1_7) exist in the *Euglena* RNA-Seq database^20^. It would be interesting to know why *Euglena* dominantly produces wax esters as a neutral lipid under anaerobic conditions, in spite of the existence of potential TAG biosynthesis ability. Further enzymlogical and physiological analyses of enzymes involved in TAG biosynthesis need to clarify this matter. With respect to lipid metabolism, it would also be crucial to understand why *Euglena* produces wax esters expressly during anaerobic fermentation. Simultaneous suppression of *WSD2* and *WSD5* genes did not affect any phenotypical and physiological parameters such as cell mobility, cell shape, cell growth, viability, and chlorophyll content, although accumulation of wax ester was reduced by approximately 70% under anaerobic conditions. On the other hand, the ratio of paramylon degradation in the double gene suppression strain under anaerobic condition showed no significant difference from that in the mock control, suggesting ATP acquisition thorough ordinary glycolysis. Thus, it is conceivable that production of wax ester is not indispensable to adapt to anaerobic conditions, and the cause and benefit of wax ester conversion under anaerobic conditions is still unclear. From the other perspective, this formidability is an important strategy for *Euglena* to survive under harsh environmental conditions^28^, even if the major route for wax ester synthesis is blocked by some unexpected reason. It might be plausible that, comparing with TAG, degradation of wax ester, which has only a single bound ester, might be the easier way to utilize the resultant products, fatty acids and alcohols, for energy generation whenever sufficient oxygen is supplied again to anaerobic environments.

In summary, we have identified WSDs that are key enzymes for wax ester synthesis in *Euglena*. We showed that *Euglena* WSDs were unique enzymes in terms of substrate specificity for the recognition of middle lengths of fatty acids and alcohols, mainly the carbon length of 14. The recombinant WSDs were functionally expressed in yeast, and development of such cell lines, perhaps coupled with FAR, might provide a promising feedstock for biodiesel and aviation biofuel.

## Materials and Methods

### Euglena strain and culture conditions

*E. gracilis* SM-ZK, a chloroplast-lacking bleached mutant derived from strain Z, was used throughout this study as it is a representative strain for previously reported wax ester research^5,6^. Cells were cultured in Koren-Hutner medium^29^ on a rotary shaker (120 rpm) under continuous light (100 µmol m^−2^ s^−1^) at 26 °C for 6 d, by which time the stationary phase was reached. Cell number was measured using the CASY Cell Counter and Analyzer System (Roche Applied Science, Basel, Switzerland).

### Cloning of Euglena WSD and WS and heterologous expression in yeast

Based on a BLASTX analysis against the *Euglena* RNA-Seq database, we identified six WSD and one WS homologs, and gene-specific primers were designed to amplify their coding region as listed in Supplementary Table S1. PCR amplification was carried out using KOD-plus polymerase (Toyobo, Japan) with *Euglena* cDNA pool as a template. All amplicons were cloned into pYES2 expression vector (Invitrogen, Carlsbad, CA) using gap-repair cloning method^30^. For yeast transformation, *S.cerevisiae* strain W303-1A was routinely grown at 30 °C in rich YPAD medium (1% yeast extract, 2% peptone, 2% glucose). The yeast was transformed using LiAc protocol^31^ with a DNA mixture containing 0.2 μg of a linearized pYES2 and 1.0 μg of amplified target fragments. The transformants were then selected on SD-drop agar plates (0.2% amino acid mixture, 0.67% yeast nitrogen base without amino acid, 2% glucose, 1.8% agar) without supplementation of the appropriate metabolite. After 3 d of incubation at 30 °C, colonies were selected for cultivation in SD-drop medium, and the plasmids were extracted, amplified in *E. coli* DH5α, and verified by DNA sequence. The complete sequence information has been deposited in the DDBJ databank under accession number LC069357 to LC069364. For heterologous expression in yeast, the resultant constructs were transformed into yeast H1246 strain, and the yeast cells were cultivated in the medium supplemented with 2% (w/v) D-galactose and various fatty acids and alcohols for 48 h at 30 °C.

### Quantitative real-time PCR experiments

*Euglena* cells grown to stationary phase were anaerobically treated for 24 h and then collected. Total RNA was prepared using a RNAiso regent (Takara). Less than 500 ng of the total RNA was used for cDNA preparation with PrimeScript RT reagent Kit with gDNA Eraser (Takara). For quantitative real-time PCR, the reaction mixture contained 2 µL of the cDNA samples (100 ng/μL), 10 µL of the SYBR Premix EX Taq (Takara), 10 µL of forward and reverse primers, and H_2_O (up to 20 µL). The reaction was run with the Light Cycler 96 system (Roche). The relative expression level normalized to malate synthase was calculated with Light Cycler Application Software. The primers used are listed in Supplementary Table 1.

### RNAi experiments

Silencing of *Euglena* WSD paralogs by RNAi was performed as described previously^32,33^. Approximately 500-bp partial *Euglena* WSD cDNAs were PCR-amplified with the addition of the T7 RNA polymerase promoter sequence at one end. The primers used are listed in Supplementary Table S1. Then the sense and antisense RNAs were synthesized using the PCR products as templates (MEGAscript RNAi Kit, Ambion). After purification of the transcribed RNA with DNase I digestion followed by ethanol precipitation, dsRNA was made by annealing equimolar amounts of the sense and antisense RNAs. *Euglena* cells of 2-d old cultures were collected and resuspended in culture medium containing 4.2 mM Ca(NO_3_)_2_, 3.7 mM KH_2_PO_4_, and 2.1 mM MgSO_4_. One hundred fifty microliters of the cell suspension (100 µL; approximately 5 × 10^6^ cells) was transferred to a 0.4-cm-gap cuvette and electroporated with 5 µl of RNA solution (15 µg of dsRNA in 50 mM Tris-HCl, pH 7.5, and 1 mM EDTA) using the NEPA21 electroporator (Nepa Gene) at 0.5 kV and 25 µF. The cell suspension was diluted with fresh KH medium and cultured at 26 °C for restoration.

### RT-PCR

Total RNA was prepared from wild-type and dsRNA-introduced *Euglena* cells using a RNAiso regent (Takara). The first strand cDNA was synthesized using a PrimeScript II 1st strand cDNA kit (Takara) with an oligo(dT) primer. The PCR was performed using specific primers as listed in supplementary Table 1. EF1-α from *Euglena* (X16890) was used as a normalizer.

### Wax ester measurement

The extraction of the total wax ester fraction from *Euglena* cells was performed according to the method described by Inui *et al*.^5^, with slight modifications. *Euglena* cells were harvested, freeze-dried, and added to 2.4 mL of a mixture of chloroform, methanol, and water in the ratio 10:20:8 (v/v/v). After thorough agitation, the mixture was centrifuged to remove cells and debris. The extraction was repeated, and the combined supernatants were added to chloroform and water (1 ml each), followed by vigorous shaking. After centrifugation, the chloroform phase was evaporated and dissolved in hexane. The wax ester extract was filtrated using the PTFE 0.22 μm filter for the GC-MS analysis. The wax ester fraction was separated and determined using GCMS-QP2010 (Shimadzu) equipped with a DB-5ms column (30 m × 0.25 mm internal diameter, 0.25 μm film thickness; Agilent Technologies). A 1-μL portion of the wax ester fraction was injected into GC-MS using a splitless injection; helium was used as the carrier gas (1.16 mL/min). Chromatographic separation was initially set at 100 °C (1 min), then the temperature was increased to 280 °C (10 °C per min), and held for 10 min. The mass transfer line and ion source were at 250 °C. Myristyl myristate was detected with electron ionization (70 eV) in the selected ion monitoring mode at m/z 229.2 and 57.1 for the quantitative analysis. All data acquisition and processing were performed with the GC-MS Solution Software (Shimadzu).

### Fatty acids measurement

*Euglena* cells were harvested, freeze-dried, and then the whole fatty acids were methyl esterified using Fatty acid methylation kit and FAME purification kit (Nacalai Tesque, Kyoto, Japan). This process converted not only free fatty acids but also bounded forms of fatty acids, such as wax ester and TAG, into FAME. The purified FAME was analyzed by GC-MS with the condition described above by using FAME compound library supplied by the manufacture.

### Thin-layer chromatography

Yeast cells were harvested and the extraction solvent chloroform/methanol/0.9% (w/v) NaCl (4:2:1, v/v/v) was added to each cell. Total lipids were then extracted from the chloroform phase after disrupting the cells with a cell disruptor (Multi-beads shocker®, Yasui kikai, Osaka, Japan). TLC plates were purchased from Merck Millipore (Glass Backed TLC Classical Silica Plate, 250 µm Thick Silica Gel 60, Darmstadt, Germany). TLC analysis was performed using the development solvent systems hexane/diethylether/acetic acid (90:7.5:1.0, v/v/v). Tripalmitin (Wako, Japan) and myristyl myristate (Sigma-Aldrich) standards were used as TAG and wax ester reference substances, respectively. The TLC plates were sprayed with a 0.005% (w/v) primulin solution in acetone:water (4:1, v/v), and lipid spots were detected by ImageQuant LAS 4010 (GE Healthcare). TAG co-migrated at the same position the standard TAG was scraped for FAME analysis.

### Lipid profile analysis

Sample preparation and subsequent lipid profile analysis were performed according to the method established by Fruhashi *et al*.^24^. In Brief, aerobically grown *Euglena* cells were placed under anaerobic conditions for 24 h. The *Euglena* cells were collected at 0 h and 24 h. Total lipids were extracted from the *Euglena* cells with MCA solvent (methanol/chloroform/2% acetic acid, 5:2:1, v/v/v), and dried in a centrifugal evaporator. Wax ester contents were determined by a GC-TOF/MS-based profiling method. For fatty acids and fatty alcohols profiling, sodium methoxide in methanol was added to the dried lipid pellet to transesterify the ester-linked lipids, followed by trimethylsilylation (TMS), then the methyl esters and the TMS derivatives were subjected to GC-TOF/MS analysis.

### Extraction and detection of paramylon

Extraction and detection of paramylon were performed as previously described by Tanaka *et al*.^34^. Paramylon contents were finally determined using the phenol-sulfuric acid method with glucose solution as a standard.

### Data analysis

The significant of differences between data sets were evaluated by the Student’s *t* tests. Calculations were carried out using Microsoft Excel software.

## Electronic supplementary material

Supplemental information
