# Supplementary material for: Wax Ester Synthase/Diacylglycerol Acyltransferase Isoenzymes Play a Pivotal Role in Wax Ester Biosynthesis in Euglena gracilis
Source: Sci Rep. 2017 Oct 18;7:13504. doi: 10.1038/s41598-017-14077-6 (PMC5647427; doi:10.1038/s41598-017-14077-6)
Supplement: Supplementary file 1 — Supplemental information [file 41598_2017_14077_MOESM1_ESM.doc]

**Wax Ester Synthase/Diacylglycerol Acyltransferase Isoenzymes Play a Pivotal Role in Wax Ester Biosynthesis in *Euglena gracilis***

Takuya Tomiyama^1,2^, Kaeko Kurihara^1,2^, Takahisa Ogawa^1,2^, Takanori Maruta^1,2^, Takumi Ogawa^3^, Daisaku Ohta^3^, Yoshihiro Sawa^1^, and Takahiro Ishikawa^1,2^,*

^1^Department of Life Science and Biotechnology, Faculty of Life and Environmental Science, Shimane University, 1060 Nishikawatsu, Matsue, Shimane 690-8504, Japan

^2^Core Research for Evolutional Science and Technology (CREST), Japan Science and Technology Agency (JST), Chiyoda-ku, Tokyo 102-0076, Japan

^3^Graduate School of Life and Environmental Sciences, Osaka Prefecture University, 1-1 Gakuen-chou, Nakaku, Sakai, Osaka 599-8531, Japan

*Corresponding. ishikawa@life.shimane-u.ac.jp

Table S1 Primer lists.

Primer name Target cDNA Experiment oligonucleotide

EgWSD1-qRT Fw EgWSD1 Quantitative-RT-PCR TCAGCCTGTATTCGTATTGTGG

EgWSD1-qRT Rv EgWSD1 Quantitative-RT-PCR GTCTGACAGGAGGCCAGTGT

EgWSD2-qRT Fw EgWSD2 Quantitative-RT-PCR ACAGCTGGCGTCCATACTTT

EgWSD2-qRT Rv EgWSD2 Quantitative-RT-PCR CAGAGCCCAACCAAGAACC

EgWSD3-qRT Fw EgWSD3 Quantitative-RT-PCR GTGAACCGCAAGCTGACC

EgWSD3-qRT Rv EgWSD3 Quantitative-RT-PCR CAAGTTGGTGCTCGTCTCTG

EgWSD4-qRT Fw EgWSD4 Quantitative-RT-PCR TGTGGGAGAAGACAGCTAAGG

EgWSD4-qRT Rv EgWSD4 Quantitative-RT-PCR GGCCAGGTAGGACTGCTTC

EgWSD5-qRT Fw EgWSD5 Quantitative-RT-PCR CTGTTCCGCTTCCACCAC

EgWSD5-qRT Rv EgWSD5 Quantitative-RT-PCR GCACAGCCCGTAGAACCA

EgWSD6-qRT Fw EgWSD6 Quantitative-RT-PCR GGGTCTCAAGACCGATTCC

EgWSD6-qRT Rv EgWSD6 Quantitative-RT-PCR GTGCAGCCCAGGGACTTAC

EgWS-pYES2 Fw EgWS pYES2 5’-cloning GCTGTAATACGACTCACTATAGGGAATATTAAGCTTATGGATTTTTTGGGGTTCCCTGAC

EgWS-pYES2 Rv EgWS pYES2 3’-cloning GCATGCTCGAGCGGCCGCCAGTGTGATGGATA TCTGTCAGACAGACAGCCCCAGCG

EgWSD2-pYES2 Fw EgWSD2 pYES2 5’-cloning GCTGTAATACGACTCACTATAGGGAATATTAAGCTTATGGTGGTAGCCGAGACC

EgWSD2-pYES2 Rv EgWSD2 pYES2 3’-cloning GCATGCTCGAGCGGCCGCCAGTGTGATGGATATCTGTCAGGTTGACGTTGTGTCC

EgWSD3-pYES2 Fw EgWSD3 pYES2 5’-cloning GCTGTAATACGACTCACTATAGGGAATATTAAGCTTATGGTTGACAGTCAGCCAGC

EgWSD3-pYES2 Rv EgWSD3 pYES2 3’-cloning GCATGCTCGAGCGGCCGCCAGTGTGATGGATATCTGTCAGATCTCGCCGCCG

EgWSD5-pYES2 Fw EgWSD5 pYES2 5’-cloning GCTGTAATACGACTCACTATAGGGAATATTAAGCTTATGGCCGTCCCAGGCATC

EgWSD3-pYES2 Rv EgWSD3 pYES2 3’-cloning GCATGCTCGAGCGGCCGCCAGTGTGATGGATATCTGCCTGAGGGCCTCGCTG

EgWSD2-RNAi Fw EgWSD2 RNAi TAATACGACTCACTATAGGGTCGGGGACCTGTTCTGG

EgWSD2-RNAi Rv EgWSD2 RNAi TAATACGACTCACTATAGGGGGCTCTCCTCGCCTTC

EgWSD3-RNAi Fw EgWSD3 RNAi TAATACGACTCACTATAGGGCAGAGACGAGCACCAACTTG

EgWSD3-RNAi Rv EgWSD3 RNAi TAATACGACTCACTATAGGGTTTGGCTTGAGGGGGC

EgWSD5-RNAi Fw EgWSD5 RNAi TAATACGACTCACTATAGGGTGGTGGCGAGTGGATG

EgWSD5-RNAi Rv EgWSD5 RNAi TAATACGACTCACTATAGGGACCAGCAGCACCTCCAG

EgWSD2-RT Fw EgWSD2 Semi-qRT-PCR CCGCCCCAATTAAGATG

EgWSD2-RT Rv EgWSD2 Semi-qRT-PCR CACAGTCTTTTGAGGCTCC

EgWSD3-RT Fw EgWSD3 Semi-qRT-PCR ACAACTGCAAAATCAACGAC

EgWSD3-RT Rv EgWSD3 Semi-qRT-PCR CAGGACATGGTCGATCG

EgWSD5-RT Fw EgWSD5 Semi-qRT-PCR GAACAAATGTCGATGCGG

EgWSD5-RT Rv EgWSD5 Semi-qRT-PCR CATGTCGTCCCACTCCG

EF1α-F EgEF1α Semi-qRT-PCR ACAGATTGGGAACGGGTACGC

EF1α-R EgEF1α Semi-qRT-PCR CGCAGTTTCCCTTCACCATCG

Underlines shows T7 promoter sequence

Semi-qRT-PCR semiquantitative qRT-PCR, RNAi RNA interference

**
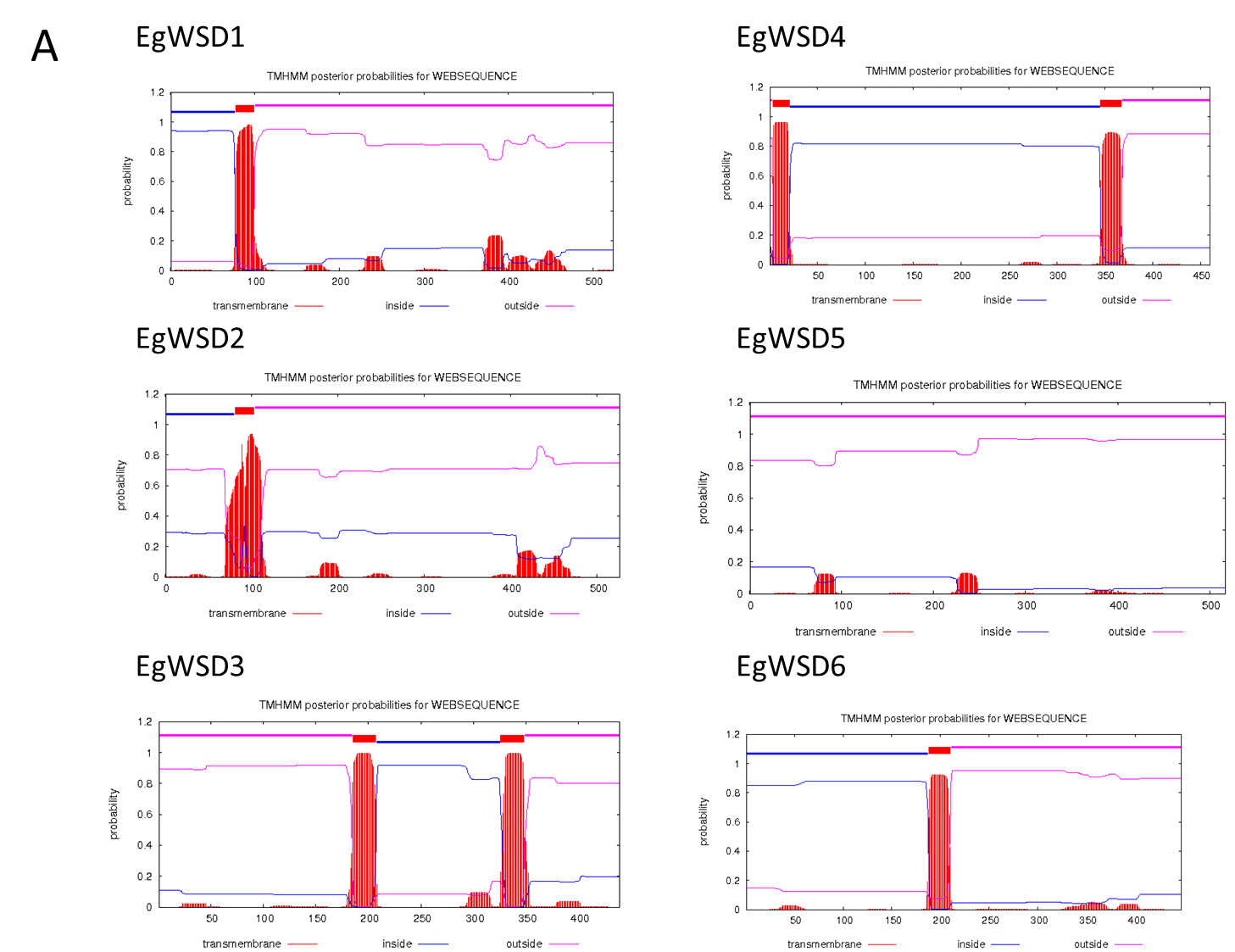
**

**
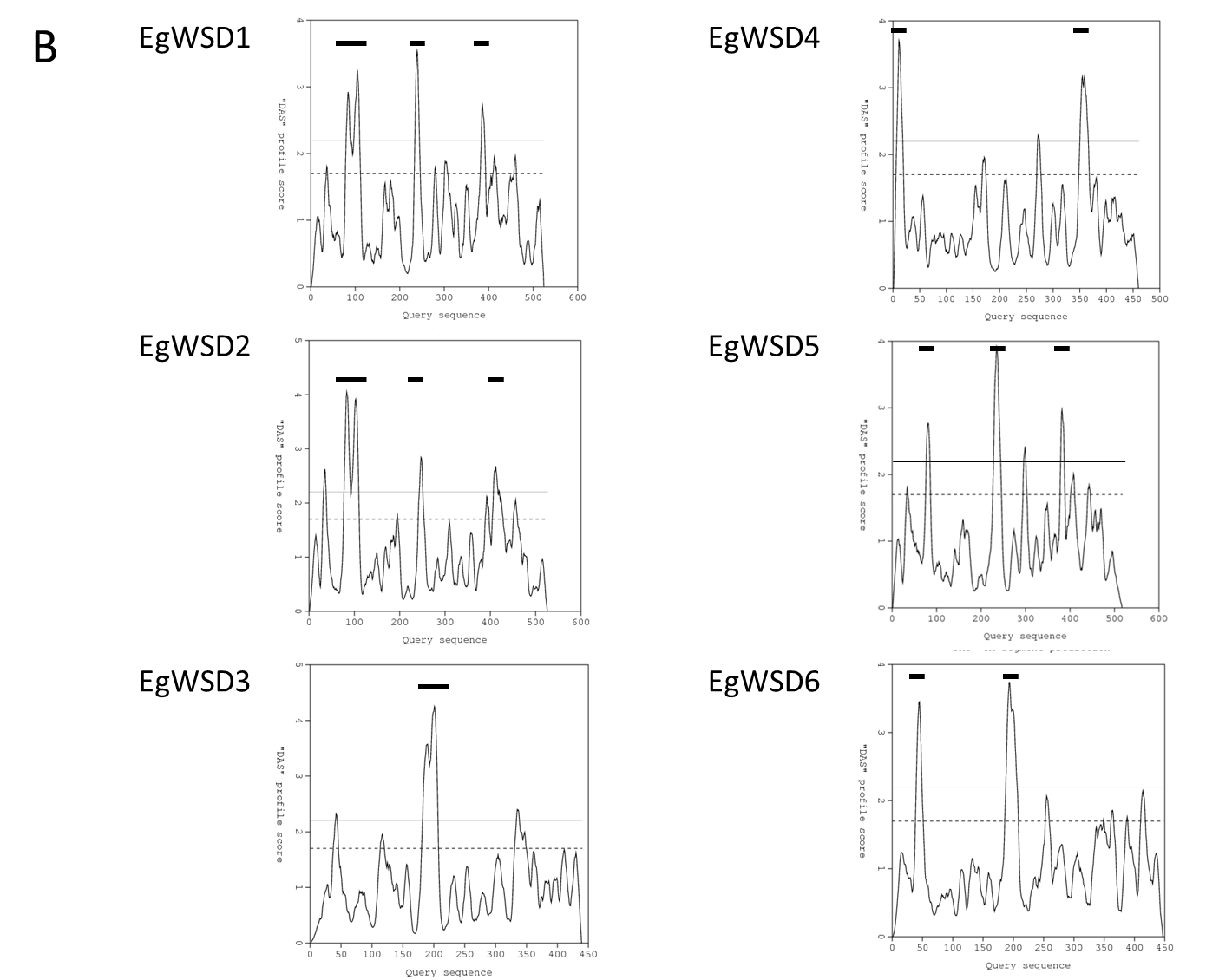
**

**
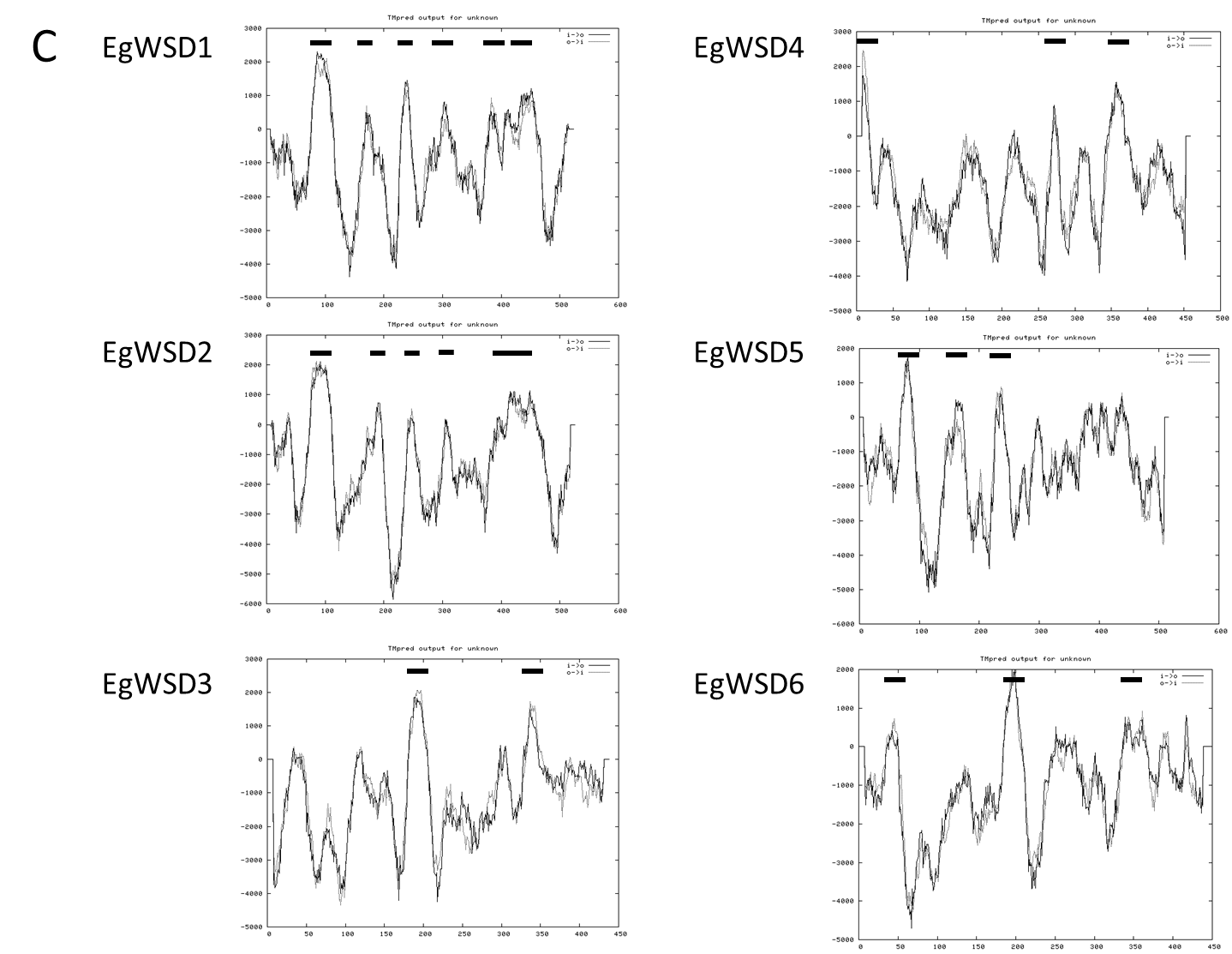
**

**
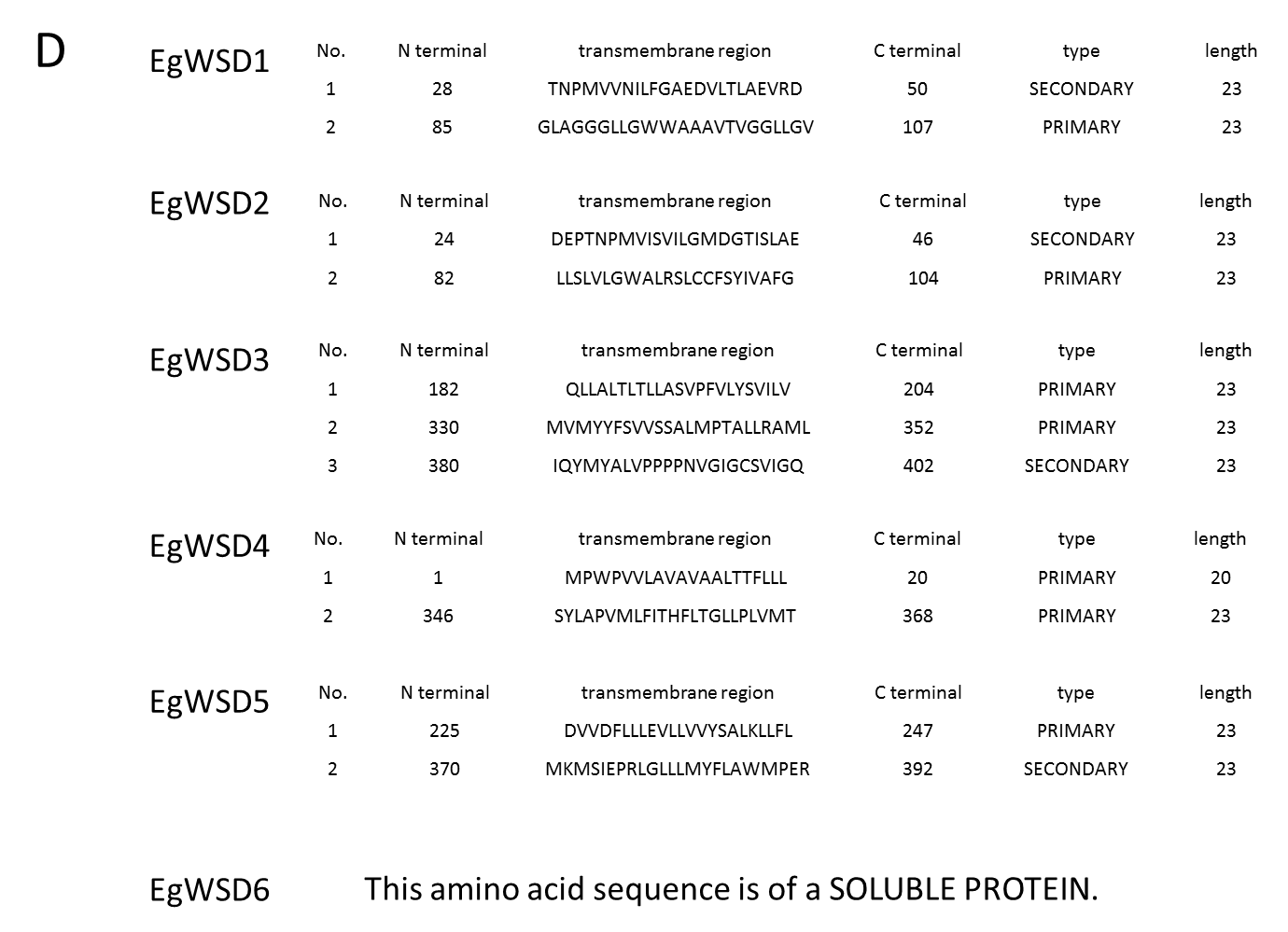
**

**Fig. S1. Transmembrane domain prediction.** (A) Putative transmembrane domains predicted by the TMHMM program (http://www.cbs.dtu.dk/services/TMHMM/). The top line shows the predicted topology with the predicted TM helices. The blue and red lines show the posterior probabilities for the inside and outside loops, respectively. The striped profile shows the probability for TM helix. (B) Putative transmembrane domains predicted by DAS-Transmembrane Prediction server (http://www.sbc.su.se/~miklos/DAS/). The top solid bar shows the predicted transmembrane domain. The solid and dot lines show the strict and loose cutoff, respectively. (C) Putative transmembrane domains predicted by TMpred (http://www.ch.embnet.org/software/TMPRED_form.html). The top solid bars show the predicted transmembrane regions. (D) Putative transmembrane domains predicted by SOSUI program (http://harrier.nagahama-i-bio.ac.jp/sosui/).


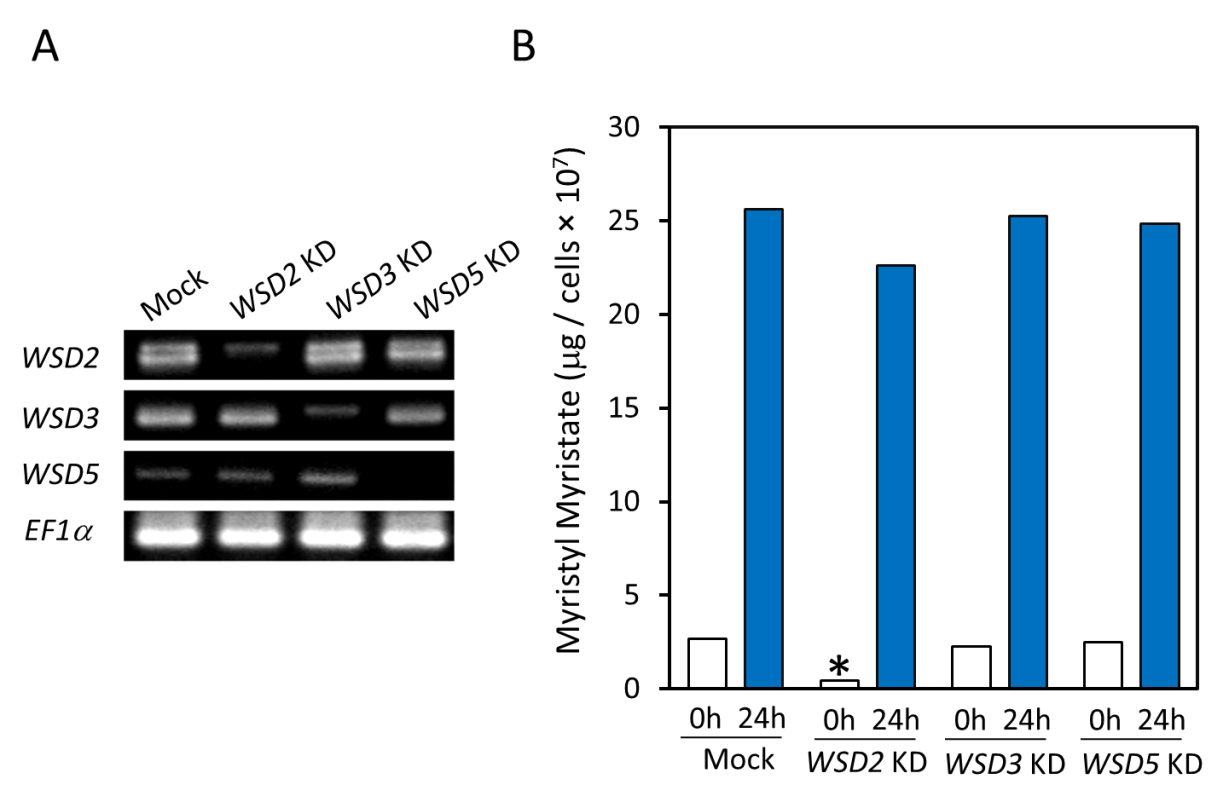


**Fig. S2. Effect of individual WSD gene knock down (KD) on myristyl myristate production.** (A) Verification of gene silencing by RT-PCR. RT-PCR was carried out using total RNA from *Euglena* cells in which dsRNA was introduced. Mock cells electroporated without dsRNA. (B) Effect of each WSD KD on myristyl myristate production under anaerobic conditions.
